# Anomalies of radial and ulnar arteries

**DOI:** 10.1590/1677-5449.011716

**Published:** 2017

**Authors:** Rajani Singh, Rashmi Malhotra, Munish Wadhawan

**Affiliations:** 1 All India Institute of Medical Sciences Rishikesh – AIIMS Rishikesh, Department of Anatomy, Uttrakhand, India.

**Keywords:** brachial artery, radial artery, ulnar artery, variation, median nerve, artéria braquial, artéria radial, artéria ulnar, variação, nervo mediano

## Abstract

During dissection conducted in an anatomy department of the right upper limb of the cadaver of a 70-year-old male, both origin and course of the radial and ulnar arteries were found to be anomalous. After descending 5.5 cm from the lower border of the *teres major,* the brachial artery anomalously bifurcated into a radial artery medially and an ulnar artery laterally. In the arm, the ulnar artery lay lateral to the median nerve. It followed a normal course in the forearm. The radial artery was medial to the median nerve in the arm and then, at the level of the medial epicondyle, it crossed from the medial to the lateral side of the forearm, superficial to the flexor muscles. The course of the radial artery was superficial and tortuous throughout the arm and forearm. The variations of radial and ulnar arteries described above were associated with anomalous formation and course of the median nerve in the arm. Knowledge of neurovascular anomalies are important for vascular surgeons and radiologists.

## Resumo

Durante dissecção de membro superior direito de um cadáver de 70 anos, do sexo masculino, conduzida em um departamento de anatomia, foram observadas anomalias tanto na origem quanto no curso das artérias radial e ulnar. Após descer 5,5 cm desde a borda inferior do músculo redondo maior, a artéria braquial anomalamente se bifurcava em uma artéria radial medialmente, e em uma artéria ulnar lateralmente. No braço, a artéria ulnar se encontrava ao lado do nervo mediano, e seguia um curso normal no antebraço. A artéria radial se encontrava medialmente ao nervo mediano no braço e, então, no nível do epicôndilo medial, ela cruzava do lado medial para o lado lateral do antebraço, na superfície dos músculos flexores. O curso da artéria radial era superficial e tortuoso em todo o braço e antebraço. As variações das artérias radial e ulnar aqui descritas foram associadas a formação e curso anômalos do nervo mediano no braço. Conhecimento sobre anomalias neurovasculares são importantes para cirurgiões vasculares e radiologistas.

## INTRODUCTION

The brachial artery is the main source of irrigation for the arm. It is the continuation of the axillary artery from the lower border of the teres major muscle. It normally terminates in the cubital fossa at the level of the neck of the radius, dividing into the radial artery laterally and the ulnar artery medially. The radial artery runs along the lateral part of the front of the forearm by the side of the superficial branch of the radial nerve. The ulnar artery passes medially deep to the pronator teres muscle and it then travels into the distal part of the forearm together with the ulnar nerve.

However, we observed an anomalously high termination of the brachial artery, dividing into radial and ulnar arteries, which also had variant courses. This resulted in shortening of the brachial artery and high origin of the radial and ulnar arteries. These variations notwithstanding, the radial artery was also tortuous. Such tortuosity would be likely to create an environment conducive to atherosclerosis,^1^ an indicator of occlusive complications. We also observed anomalies in the origin and course of the ulnar artery in the arm. These variations were found in combination with the anomalous formation and course of the median nerve. This present case, with so many variations, therefore has important clinical implications for vascular surgeons and radiologists, and is also essential reading for anatomists interested in rare and unique variations.

## CASE REPORT

**Figure 1:**
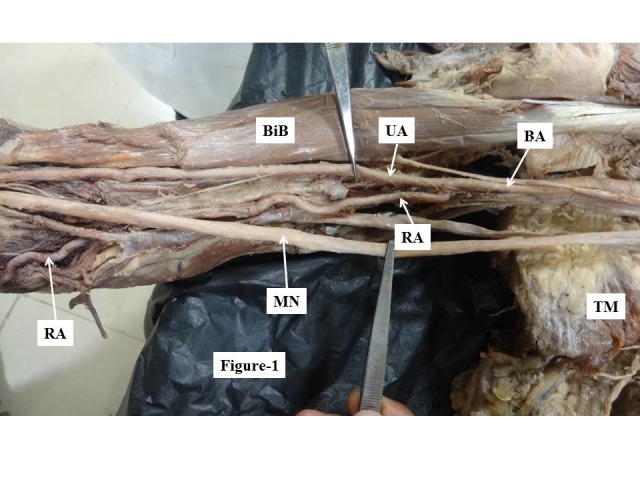
Showing high bifurcation of the brachial artery into radial and ulnar arteries and an abnormal course of the median nerve in the arm. MN = median nerve; RA = radial artery; UA = ulnar artery; BA = brachial artery; BiB = biceps brachii muscle; TM = teres major muscle.

**Figure 2:**
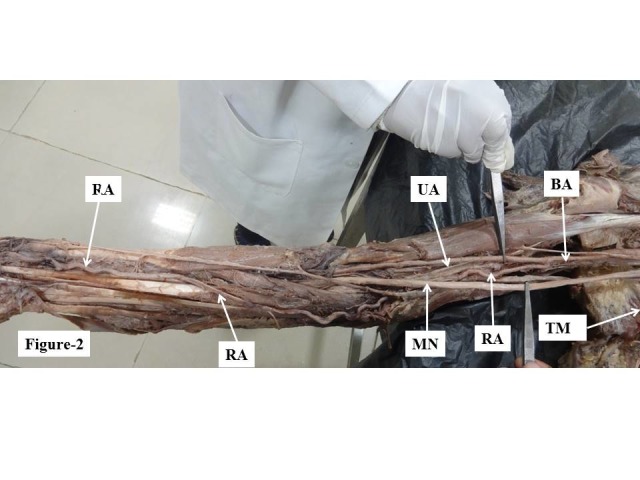
Showing tortuous course of the radial artery in the arm and forearm. MN = median nerve; RA = radial artery; UA = ulnar artery; BA = brachial artery; TM = teres major muscle.

During a dissection of the right upper limb of the cadaver of a 70-year-old male fixed in 10% formalin that was conducted in an anatomy department, we observed an anomalously high origin from the brachial artery of the radial and ulnar arteries, which also had variant courses. The brachial artery bifurcated into the radial artery medially and the ulnar artery laterally at 21 cm cranial of the cubital fossa (Figure 1), whereas normally it divides at the cubital fossa itself with a different disposition. After descending 5.5 cm below the lower border of the teres major muscle, and at 21 cm above the cubital fossa, the brachial artery terminated anomalously, giving rise to the radial artery medially and the ulnar artery laterally. The radial artery remained medial to the median nerve in the arm (Figure 1). At the elbow, the radial artery continued along the medial aspect and then crossed to the lateral side at the level of the upper third of the forearm, superficial to the flexor muscles (Figure 2). The radial artery was found to be tortuous from the beginning to around the medial epicondyle. Thereafter it ran straight along its oblique course, before once again becoming highly tortuous. The total length of the radial artery was 55 cm. The ulnar artery remained on the lateral side of the arm, then entered the forearm by passing between two heads of the flexor carpi ulnaris muscle and remained on the medial aspect of the forearm. These anomalies were associated with abnormal formation and course of the median nerve. The lateral root crossed the axillary artery anteriorly and fused with the medial root to form the median nerve on the medial aspect (Figure 1). It remained on the medial aspect and in the cubital fossa it traversed between the two heads of the pronator teres, entered the forearm and followed the normal course.

## DISCUSSION

Normally, the brachial artery bifurcates into the radial and ulnar arteries at the level of the neck of the radius, in the cubital fossa. Variations in the origin and course of radial and ulnar arteries are not uncommon and have been described by various authors. However, here we did not only observe a very high division of the brachial artery into radial and ulnar arteries, at 21 cm above the cubital fossa (Figure 1), combined with abnormal courses of these vessels and severe tortuosity in the radial artery, we also found these features in association with a variant origin and course of the median nerve, which make the case more interesting.

Several authors^2^^-^ 5 have reported a high division of the brachial artery, similar to our study. However, our case differs from those described by other authors in terms of disposition, course, and presence of tortuosity in the radial and ulnar arteries.

Satyanarayana et al.^2^ describe a case in which the brachial artery divided into a lateral radial artery and a medial ulnar artery, in contrast with our case, in which the brachial artery bifurcated into a radial artery medially and an ulnar artery laterally. Moreover, in our case the radial artery was tortuous, in contrast to that described by Satyanarayana et al.^2^ Also, our case includes a variant formation and course of the median nerve, whereas in their study the course of the median nerve was normal.

Guha and Palit^3^ described a case in which the brachial artery divided into radial and ulnar arteries in the middle of the arm, combined with a variant median nerve and an absent musculocutaneous nerve, unlike our case, in which the division of the brachial artery occurred in the upper third of the arm. In our case, the musculocutaneous nerve was present as normal, but was associated with an abnormal formation and course of the median nerve, unlike the case described by Guha and Palit.^3^

In a case described by Shetty et al.^4^ the brachial artery divided into the radial and ulnar arteries in a manner similar to that described in our study. However, the radial artery crossed the ulnar artery and median nerve in the arm, in contrast to our case, in which the radial artery crossed to the lateral side below the elbow. Also, in our case the median nerve lay lateral to the radial artery, but medial to the ulnar artery, while in that case the median nerve was lateral to the ulnar artery.^4^

Jayasabarinathan et al.^5^ also observed high bifurcation of the brachial artery into radial and ulnar arteries. In that case, the radial artery was not tortuous and the radial artery and ulnar artery were lateral and medial, respectively, unlike in our case.

Higher divisions of the brachial artery were noted by Bidarkotimath et al., in two cases in which the brachial artery bifurcated in the middle of the arm into radial and ulnar arteries,^6^ unlike our case, in which the brachial artery bifurcated into radial and ulnar arteries in the upper arm.

A brachial artery dividing into a lateral branch that continued as a radial artery and a medial branch continuing as an ulnar artery has also been described.^7^ However, our case differs in that the radial artery was located medially and the ulnar artery laterally. Also, in our case the brachial artery bifurcated in the upper part of the arm, while in the above case it bifurcated in the middle of the arm.

### The embryological basis of high origin of radial and ulnar arteries

The lateral branch of the seventh intersegmental artery (subclavian) is known as the axis artery of the upper limb-bud. The proximal part of the main trunk forms the axillary artery which continues as the brachial artery and its distal part persists as the anterior interosseous artery. The radial and ulnar arteries are the last arteries to appear in the forearm. At first, the radial artery arises more proximal than the ulnar artery from the main trunk and crosses in front of the median nerve. Later, the radial artery establishes a new connection with the main trunk at or near the level of origin of the ulnar artery. The upper portion of its original stem usually disappears. Thus, the radial and ulnar arteries arise at the same level. In this case, the proximal origin of the radial artery fails to disappear and the radial artery does not establish a new connection with the main trunk near the origin of the ulnar artery. The radial artery therefore originates at a higher level,^8^ as in our case.

### Clinical significance

A superficial course of the radial artery, as seen in our case, may be more vulnerable to trauma, and thus to hemorrhage. It may also lead to puncture of the radial artery during venepuncture, misinterpretation of angiographic images, or severe disturbances of hand irrigation during surgical procedures on the arm.^9^ A high brachial artery bifurcation may pose difficulties for percutaneous brachial artery catheterization techniques. This variation may cause difficulties while measuring blood pressure. It is also prone to damage during orthopedic and plastic surgeries. Tortuous arteries are favored sites of atherosclerosis. The radial artery is commonly used for coronary grafting. Lack of awareness of this variant of the radial artery, as seen in our case, could lead to failure of this procedure.

Furthermore, the abnormal course of the median nerve could lead to it being damaged during endovascular surgery and repair of peripheral nerves in the arm.

Therefore, variations including abnormal division of the brachial artery and a superficial course of the radial nerve, associated with an abnormal course of the median nerve, are of paramount importance to vascular surgeons, physicians, and radiologists when interpreting angiographs.
